# Fast and efficient isolation of murine circulating tumor cells using screencell technology for pre-clinical analyzes

**DOI:** 10.1038/s41598-024-66032-x

**Published:** 2024-07-01

**Authors:** Fei Ye, Janine Wechsler, Amira Bouzidi, Georges Uzan, Sina Naserian

**Affiliations:** 1 ScreenCell, 62, Rue de Wattignies, 75012 Paris, France; 2 INSERM UMR-S-MD 1197, Hôpital Paul Brousse, Villejuif, France

**Keywords:** Circulating tumor cells, Liquid biopsy, Precision medicine, Biomarker, Preclinical model, Biomarkers, Cancer, Oncology

## Abstract

Circulating tumor cells (CTCs) represent a rare and heterogeneous population of cancer cells that are detached from the tumor site and entered blood or lymphatic circulation. Once disseminated in distant tissues, CTCs could remain dormant or create a tumor mass causing serious danger for patients. Many technologies exist to isolate CTCs from patients’ blood samples, mostly based on microfluidic systems or by sorting them according to their surface antigens, notably EpCAM, and/or cytokeratins for carcinoma. ScreenCell has developed an easy-to-use, antigen-independent, rapid, cost-effective, and efficient technology that isolates CTCs according to their bigger size compared to the blood cells. This study provides the technical information necessary to isolate and characterize CTCs from mouse blood. By using blood samples from transgenic mice with breast cancer or from WT mice in which we spiked cancer cells, we showed that ScreenCell technology is compatible with standard EDTA blood collection tubes. Furthermore, the ScreenCell Cyto kit could treat up to 500 µl and the ScreenCell MB kit up to 200 µl of mouse blood. As the ScreenCell MB kit captures unaltered live CTCs, we have shown that their DNA could be efficiently extracted, and the isolated cells could be grown in culture. In conclusion, ScreenCell provides a rapid, easy, antigen-independent, cost-effective, and efficient technology to isolate and characterize CTCs from the blood samples of cancer patients and murine models. Thanks to this technology CTCs could be captured fixed or alive. Murine cancer models are extensively used in pre-clinical studies. Therefore, this study demonstrates the crucial technical points necessary while manipulating mouse blood samples using ScreenCell technology.

## Introduction

Metastasis is one of the main hallmarks of cancer and the reason for most cancer-related mortalities^1,2^. This process occurs when cancer cells break away from the original site and reach secondary distant tissues or organs. Metastases pose a significant challenge in cancer treatment, as they can invade vital organs and disrupt their normal functions. They can also cause additional symptoms and complications, making the management of metastatic cancer more complex^3^.

Circulating tumor cells (CTCs) are cancer cells that have detached from a primary tumor and entered the bloodstream or lymphatic system in a process that is called intravasation^4^. Through their adhesion to endothelial cells, a small population of CTCs with a more invasive profile will then extravasate in distant tissues where they remain dormant or form secondary tumor mass^5^. The microenvironment at the secondary site plays a crucial role in supporting or inhibiting the growth of metastatic tumors. Factors such as the presence of growth factors and immune cells in addition to angiogenesis, and extracellular matrix can influence the survival, proliferation colony formation of CTCs^6^. Similarly, the properties of the tumor cells, such as their ability to invade and adapt to new environments contribute to the success of metastasis^7^.

CTCs can enter blood circulation in a single cell or cluster form (two or more cells)^8^. CTC clusters are believed to better survive shear stress and anoikis and also to escape from the immune response mostly through their adhesion to platelets^9–11^. Indeed, the presence of CTC clusters is associated with increased metastatic potential and a worse prognosis in cancer patients^12,13^.

Primary tissue biopsies are the gold standard for cancer diagnosis and designing treatment strategies. Yet, they are invasive, costly, incapable of representing tumor heterogeneity, and sometimes difficult to access, especially in more profound cancers^14–16^. Liquid biopsy, however, allows non-invasive, fast, repeatable, real-time, and efficient evaluation of the tumor entities. They include circulating tumor DNA (ctDNA), extracellular vesicles (EVs), and especially CTCs within the patient's blood samples or any other body fluids^17^.

Nevertheless, CTCs are extremely rare (around one cell among millions of leukocytes) in blood circulation and this makes their capture and analysis challenging. Several technologies have been developed to isolate CTCs mostly based on complex or expensive microfluidic systems and/or targeting epithelial markers such as epithelial cell adhesion molecule (EpCAM) and cytoskeletal proteins like cytokeratin (CK)^18,19^. Although immunological approaches seem to be efficient, several studies demonstrated that CTCs of carcinoma origin have a heterogeneous expression of EpCAM and/or CK markers especially when they are in epithelial to mesenchymal transition (EMT) status. EMT is associated with improved CTC migratory, invasive properties, and inefficiency in their capturing leads to ignorance of the most aggressive CTC population^20^.

ScreenCell®, however, offers a simple, rapid, and disposable solution to isolate CTCs based on microfiltration technology^21^. Using ScreenCell Cyto kit for fixed cells or MB kit for unaltered live cells, CTCs are captured based on their bigger size compared to leucocytes in the patient's blood. Thanks to its independency over any machinery or antibodies-antigen interaction, ScreenCell technology remains cost-effective and accessible to anyone interested in liquid biopsy and precision medicine investigations and clinical applications.

Although clinical trials are necessary steps to validate the final safety and utility of CTCs as a liquid biopsy, pre-clinical models, especially, mouse models provide a crucial system to assess the hypothesis flourished from in vitro experiments. Mouse models of cancers have played a pivotal role in our better understanding of metastatic cancer biology^22^. Yet, the current technologies for CTC isolation in mice are not satisfying enough for efficiently capturing CTCs and for a variety of experimental conditions and optimal results.

This study provides a comprehensive description of CTC isolation from mouse blood using ScreenCell technology. It clarifies the optimal conditions of (1) the blood collection tube compatible with mouse blood, (2) the volume of mouse blood that could be treated by ScreenCell kits, and (3) the maximum time that blood could be preserved before starting the CTC isolation. Additionally, using transgenic PyMT mouse models that spontaneously develop breast cancer (BC), also by spiking mouse BC cell line (4T1) and human BC cell line (MCF7) into healthy WT mouse blood, we isolated single and cluster forms of CTCs, showed their clear cell morphology and distinguished them from normal epithelial cells. Furthermore, since ScreenCell provides access to live CTCs, we evaluated the possibility of having access to their genomic DNA and eventually growing these cells in culture for further applications.

## Methods

### Animal models and blood collection

All animals were housed under specific pathogen-free conditions at the animal facility of Inserm-UMS44 located at Paul Brousse Hospital, Villejuif, France. The health status of mice was monitored regularly by animal facility staff. The local temperature ranged from 23 to 25 °C, the humidity from 40 to 60%, and 12/12 h of light/dark cycles were respected. The mice were housed with 3–5 animals in individually ventilated cages, with adequate access to a normal chow diet and water. All the required conditions are validated according to the ethical committee: CAPSud—Comité d'éthique CEEA n°26.

To collect normal mouse blood, we used 8 to 14-week-old female wild-type C57BL/6 mice (30 in total) (Envigo, Gannat, France). Sterile glass microcapillary tubes (Fisher Scientific, Illkirch, France) were first rinsed with PBS-EDTA solution (1.8 mg/ml final concentration). Then, immediately after sacrificing the mice with cervical dislocation, the maximum amount of blood was either drawn via cardiac puncture manipulation or retro-orbitally from the sinus of each mouse until reaching the final required blood volume. All animal experimental procedures throughout this project have been validated by the animal welfare structure (la structure du bien être animal (SBEA)) of the animal facility and have been carried out under good ethical and animal welfare practices.

PyMY transgenic mice have been bred locally under the already described standard conditions. Upon the appearance of a breast tumor, mice (5 in total) were anesthetized by isoflurane gas and sacrificed through cervical dislocation, and around 1 ml of blood was collected via cardiac puncture manipulation. Immediately after collection, blood was transferred into Heparin (Sarstedt, nümbrecht Germany) or K2-EDTA vacutainer (Becton Dickinson, Mississauga, Canada).

### Tumor cell line preparation

4T1 mouse breast cancer cell line (passages 12–15) and MCF7 human breast cancer cell line (passages 7–10) were received from Inserm U1197 (Paul Brousse Hospital, Villejuif, France) and cultured in Dulbecco’s modified Eagle medium (DMEM) (Gibco, Life Technologies, USA) supplemented with 10% FBS (Pan biotech, Aidenbach, Germany) and 1% Penicillin/Streptomycin (P/S) (Thermofisher Scientific, Waltham, MA, USA). Upon 80% con-fluency, cells were detached using trypsin–EDTA (Thermofisher Scientific, Waltham, MA, USA), counted manually using trypan blue, and diluted in required numbers in PBS 1X (Thermofisher Scientific, Waltham, MA, USA). Cells were then directly spiked in collected mouse blood for further experimental procedures.

### CTC isolation using ScreenCell Cyto kit

ScreenCell® Cyto devices (ScreenCell, Paris, France) were used to isolate single and cluster CTCs. Briefly, different volumes of whole blood (50, 100, 200, 500, and 1000 µl) were diluted with PBS-EDTA solution and completed to 3 mL. Then, the diluted blood sample was incubated with 4 ml of ScreenCell FC2 fixation buffer to allow red blood cells (RBCs) lysis and the preservation of the nucleated cells during 8 min of incubation time at room temperature. After that, diluted blood samples were transferred to the ScreenCell Cyto device which holds an isolation support (IS) with an 18-µm-thick polycarbonate membrane with circular pores (6.5 ± 0.33 µm) distributed randomly throughout the membrane. The diluted blood is then drawn through the membrane by vacuum force and 1.6 ml of PBS 1X (Thermofisher Scientific, Waltham, MA, USA) was added at the end of the filtration to clean the rest of the blood waste. Subsequently, the IS was released from the ScreenCell Cyto device, rinsed with PBS 1X, dried on absorbent tissue, and colored with RAL555 (Cat# 720-0351, VWR, International, Radnor, Pennsylvania, USA) for cytomorphological analysis. The characterization and enumeration of CTC were performed by an experienced cytopathologist (JW) who was unaware of the histological diagnosis using a NIKON eclipse 80i microscope integrated with a cooled CCD camera system and NIS-Elements BR2.30 imaging software (Nikon, Tokyo, Japan). Captured cells were classified as CTC if all 3 of the following cytological criteria were present: 1) Nuclei larger than 3 times the calibrated 6.5 μm ScreenCell IS pore size; 2) Irregular nuclear outline; and 3) High nuclear/cytoplasmic ratio.

### CTC isolation using ScreenCell MB kit

Different volumes of mouse blood samples (20, 50, 100, 200, 500 µl) were diluted with PBS-EDTA solution, completed to 6 mL, and incubated with 1 ml of ScreenCell LC dilution buffer for 2 min at room temperature. The enrichment of CTCs was performed using the ScreenCell MB device. At the end of the enrichment step, the MB IS was cleaned from the blood waste using 1.6 ml RNase-free PBS 1X (Thermofisher Scientific, Waltham, MA, USA), and released into a 1.5 ml microcentrifuge tube for further DNA extraction procedures.

### ScreenCell technology recovery rate

To evaluate the recovery rate of ScreenCell technology, either 0, 5, or 10 cells were precisely picked under the microscope and then spiked into 100 µl of blood samples of healthy WT mice. ScreenCell Cyto kit (ScreenCell, Paris, France) was then used to treat these blood samples as explained previously. Subsequently, the ISs were colored with RAL555 (Cat# 720-0351, VWR, International, Radnor, Pennsylvania, USA) for cytomorphological analysis. The enumeration of CTC was performed using a NIKON eclipse 80i microscope (Nikon, Tokyo, Japan).

### DNA extraction and quantification

Genomic DNA was isolated from the cells captured on the MB kit IS using the QIAamp DNA Micro Kit, Cat. No. / ID: 56304 (Qiagen, Hilden, Germany). 105 µl of lysis buffer was added to the MB kit IS. The IS was then incubated at 56 °C for 10 min and centrifuged at 12000 g for 1 min. The DNA extraction was pursued following the manufacturer’s protocol. Genomic DNA was eluted in 25 µl of nuclease-free water (Thermo Fisher Scientific, Waltham, MA, USA).

The concentration of DNA was determined using Qubit 1X dsDNA HS Assay Kit (high sensitivity, 0.1–120 ng) with a Qubit 4.0 fluorometer (Thermo Fisher Scientific, Waltham, MA, USA) according to the manufacturer’s protocols. A sample volume of 1 μl was added to 199 μl of a Qubit working solution.

### CTC culture using ScreenCell MB kit

100 µl of WT mouse blood samples containing 500 spiked 4T1 cancer cells were diluted with PBS-EDTA solution to complete to 6 ml, and then, incubated with 1 ml LC dilution buffer for 2 min at room temperature. At the end of incubation, 1.6 ml of DMEM medium containing 10% FBS and 1% P/S was added to stop the lysis procedure, and the enrichment was performed using the ScreenCell MB device. Afterward, the IS was directly released into a 24-well tissue culture plate. The CTCs were grown for 10 days in a humidified atmosphere of 5% CO2 at 37 °C using DMEM supplemented with 10% FBS (Pan biotech, Aidenbach, Germany) and 1% P/S (Thermofisher Scientific, Waltham, MA, USA). The medium was changed every 2 days.

## Results

### ScreenCell technology is based on a simple and rapid microfiltration system

**Figure 1:**
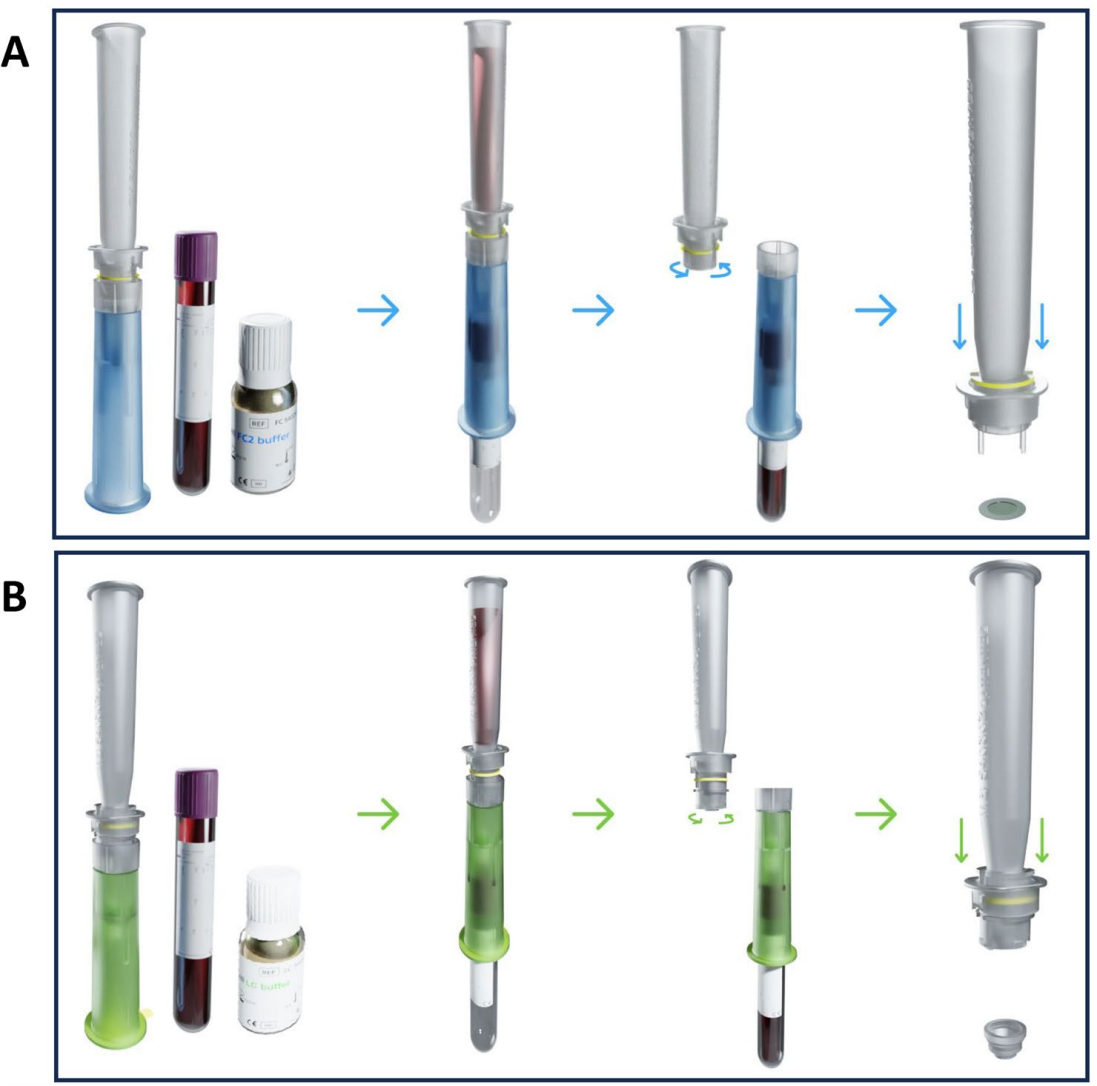
This figure depicts the technical structure of ScreenCell kits. (**A**) ScreenCell Cyto kit is dedicated to the isolation of fixed CTCs for further cytological and immunological analyses. (**B**) ScreenCell MB kits are dedicated to the isolation of unaltered live CTCs for further molecular biology and culture purposes.

ScreenCell technology has been designed and developed to enrich CTCs from any body fluids, particularly from the blood of cancer patients based on their bigger size compared to leucocytes. ScreenCell has developed two kits for different downstream applications but with similar methodological principles. ScreenCell Cyto kit is dedicated to the cytological and immunological analysis of CTCs. While using human samples, this kit could filter up to 3 ml of blood that should be mixed with ScreenCell FC2 buffer that fixes the cells and lyses the red blood cells (RBCs). To initiate the filtration process, diluted blood is added to the container and an empty vacuum blood collection tube is inserted from the bottom of the device to create a depression force. Within around 3 min, blood will pass from the container through the isolations support (IS) located in the middle of the device and will be collected in the inserted vacuum tube. In this setting, the bigger cells (CTCs and some remaining large leucocytes) will be retained on the filter. Once the filtration process is finished, the IS could be simply ejected by first rotating the upper part of the device and pushing down the IS releasing handles located on the bilateral sides of the device (Fig. 1A).

ScreenCell MB kit is dedicated to molecular biology analysis. It is based on the same technical principle as the Cyto kit. However, this kit is designed to isolate unaltered live CTCs, therefore, blood is mixed with ScreenCell LC buffer that does not contain any fixative agent and only partially lyses the RBCs. While working with human samples, the ScreenCell MB kit could filter up to 6 ml of blood. Its last difference is the shape of the MB kit’s IS which is in the form of a capsule with a smaller membrane surface (Fig. 1B). This capsule is designed to be inserted into 1.5 ml microcentrifuge tubes for proceeding to DNA or RNA extraction. Moreover, the IS capsule could be directly inserted into P24 well plates for cell culture purposes.

### The choice of collection tube and the optimal volume for mouse CTC isolation

**Figure 2:**
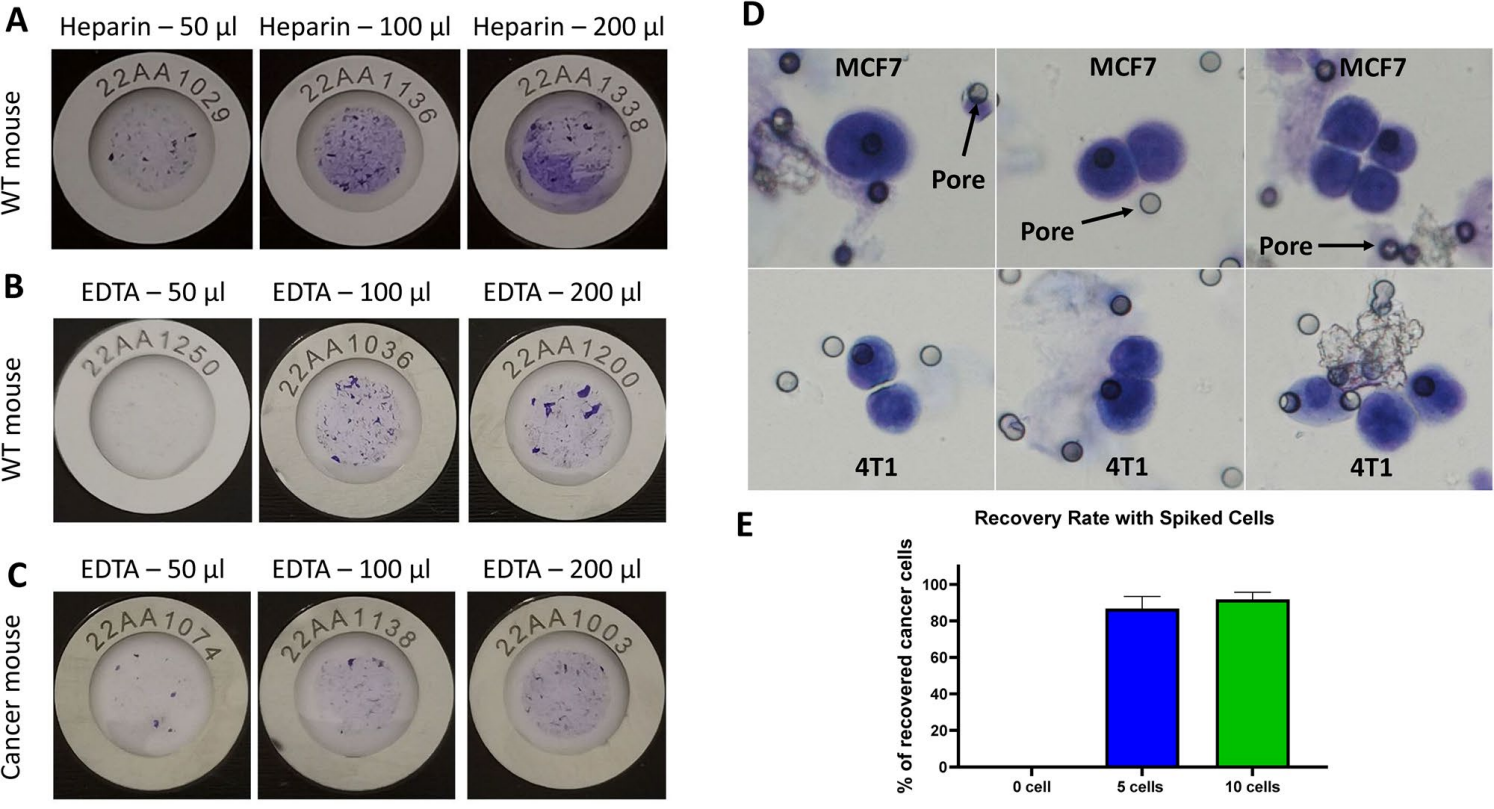
Different volumes of WT mice blood collected either within (**A**) heparin tubes or (**B**) EDTA tubes were filtered with ScreenCell Cyto kits. Similar volumes of PyMT cancer mice blood were also processed with ScreenCell Cyto kits (**C**). To evaluate the correct morphology of cancer cells in EDTA tubes, MCF7, and 4T1 breast cancer cell lines were spiked into WT mice blood and processed with ScreenCell Cyto kits. The images were taken using 40X magnification (**D**). To evaluate the recovery rate of ScreenCell technology, either 0, 5, or 10 MCF7 cells were spiked into 100 µl of blood samples of healthy WT mice. Captured cancer cells were counted on Cyto ISs and recovery rate was calculated in percentage. Data are presented as mean ± SEM (n = 6) (**E**). All experiments were repeated at least 2 times.

The main purpose of this article is to describe the optimal conditions for isolating CTCs from mouse blood samples. Therefore, we have first aimed to find out the best choice of blood collection tube containing different anticoagulants that are compatible with our technology. The second principal aim was to evaluate the maximum volume of mouse blood that could be proceeded with ScreenCell technology. As common choices for human studies, we have selected heparin and EDTA tubes. In this setting, we observed a clear difference between those two choices since the Cyto IS were much cleaner and easier to analyze while using EDTA tubes compared to heparin tubes (Fig. 2A,B). Additionally, using different blood volumes, we showed that the ScreenCell Cyto kit could perfectly filter from 50 to 200 µl of blood from healthy WT mice. Our effort to increase this volume led to the successful filtration of up to 500 µl but the blockade of the device using 1 ml of blood (data not shown). To validate these results in cancer conditions, we used different volumes of blood from either transgenic PyMT mice with spontaneous BC (referred to as cancer mouse) or by spiking MCF7 human BC cell line and 4T1 mouse BC cell line into the blood of WT mice. Our data showed perfect filtration in cancer conditions using either 50, 100, or 200 µl of blood (Fig. 2C). Moreover, the spiked MCF7 and 4T1 cells were completely intact and detectable while using ScreenCell Cyto kits (Fig. 2D). Finally, to evaluate the recovery rate of ScreenCell technology, we spiked 0, 5, and 10 MCF7 cells into 100 µl of blood from healthy WT mice and counted the captured BC cells. Our results showed that while there were no cells in blank condition, the recovery rate for 5 cells and 10 cells was 86.6% and 91.6%, respectively (Fig. 2E). Thus, a K2-EDTA vacutainer whose concentration of EDTA is consistent with ScreenCell FC2 buffer is recommended to preserve mouse blood and using the ScreenCell Cyto device one can filter up to 500 µl of mouse blood.

### Using EDTA tubes, mouse blood could be kept for up to 24 h

**Figure 3:**
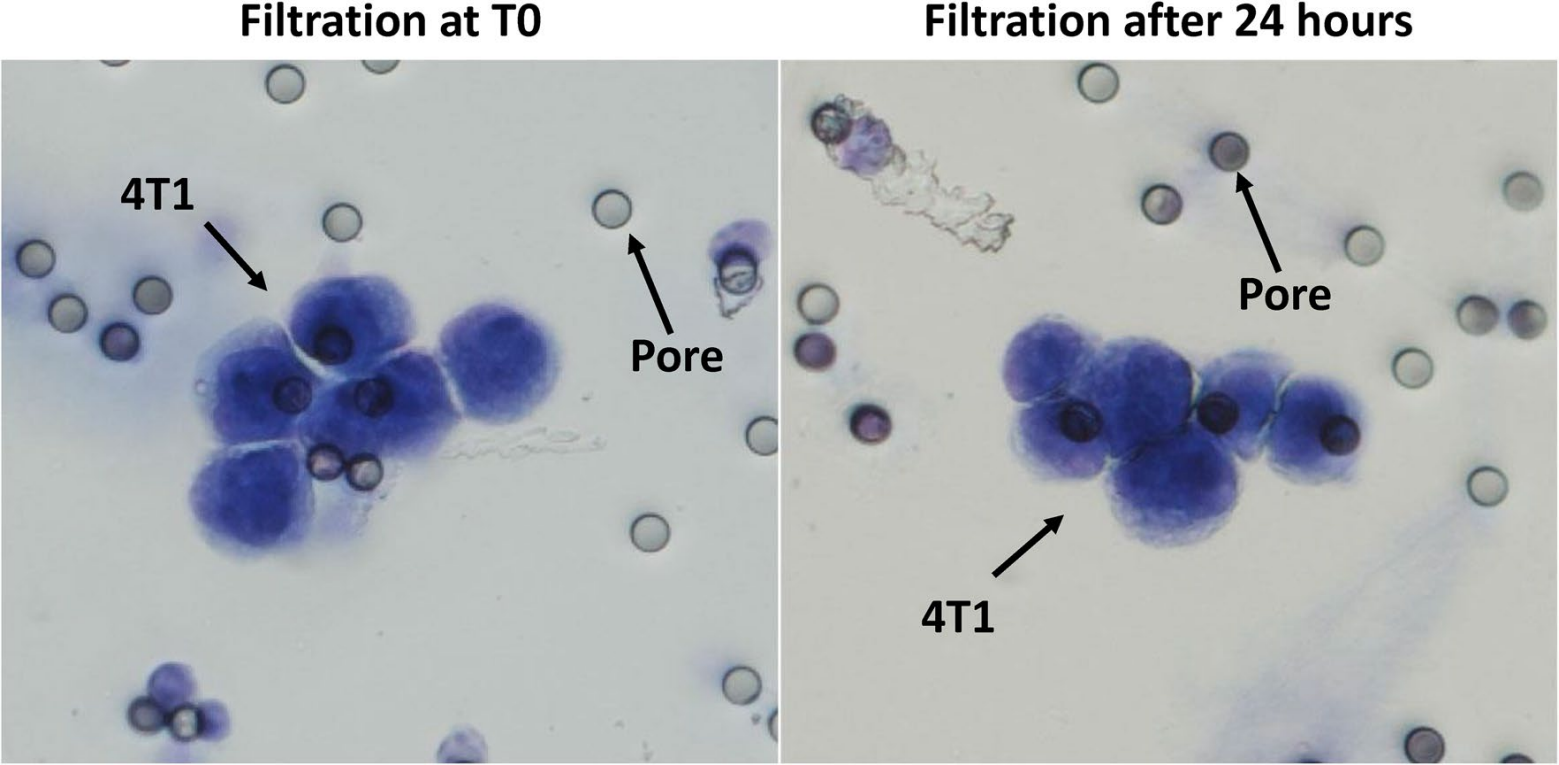
This figure represents the 4T1 cancer cells isolated using ScreenCell Cyto kits either immediately after the blood draw (left image, 40X) or after 24 h of preservation at 4 °C in EDTA tubes (right image, 40X). This experiment was performed 4 times. T0: time zero.

A normal manipulation range of EDTA tubes for human blood samples is up to 4 h. Here, we sought to investigate if the same principle applies to mouse blood samples. Thus, 1000 4T1 tumor cells were spiked into samples of 100 µl of WT mice blood. Then, blood samples were either treated immediately or were kept at 4 °C for 24 h. Interestingly, we observed that in both conditions blood samples were correctly filtered and cancer cells were perfectly preserved (Fig. 3). Any attempt to increase this period led to abnormal cell morphology (data not shown).

### ScreenCell technology enables the isolation of single and cluster CTCs

**Figure 4:**
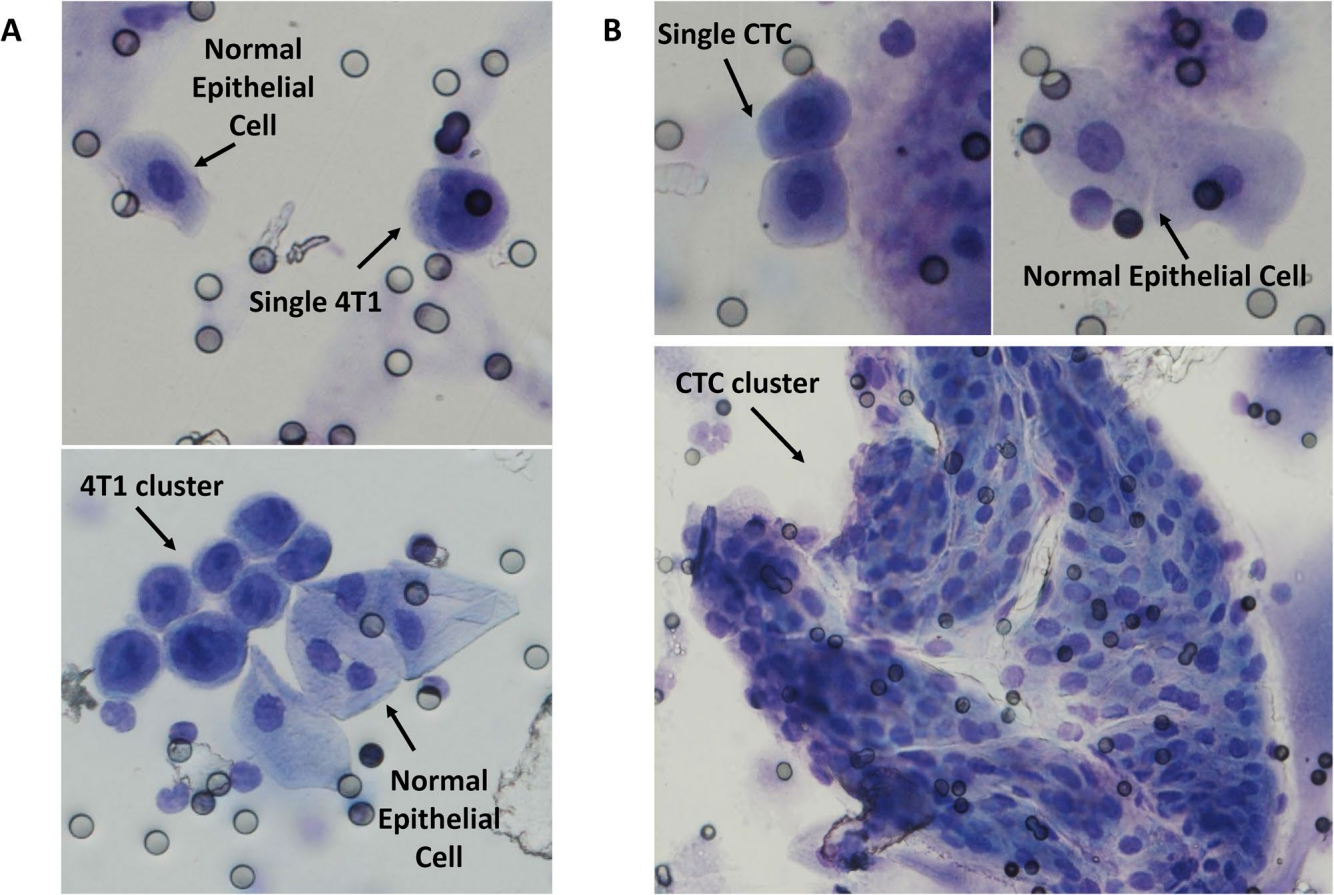
ScreenCell Cyto kits can isolate both single and cluster CTCs. (**A**) After spiking into WT mice blood, both single and cluster 4T1 cells were captured by ScreenCell Cyto kits. (**B**) Single and cluster CTCs were also efficiently captured from breast cancer mice blood. In all conditions, CTCs were distinguishable from normal epithelial cells according to their morphological features. These experiments were performed at least 3 times. All images are taken using 40X magnification, except for the CTC cluster (lower right) which is in 20X.

Few technologies are capable of providing access to both single and cluster CTCs. We have used two conditions for evaluating this crucial capability: (1) by spiking 4T1 BC cells into healthy WT mouse blood and (2) by using blood from the PyMT BC model. We have been successfully able to isolate both single and cluster CTCs whether by using a 4T1 cancer cell line (Fig. 4A) or using blood samples of mice with BC (Fig. 4B). Indeed, the procedure of blood drawing could lead to normal tissue injuries and the entering of normal epithelial cells into blood samples. Interestingly, our further cytological analysis made a clear morphological difference between normal epithelial cells and cancer cells, particularly according to their nucleus-to-cytoplasmic ratio (Fig. 4A,B).

### ScreeCell technology enables the isolation of live CTCs and access to their genetic material

**Figure 5:**
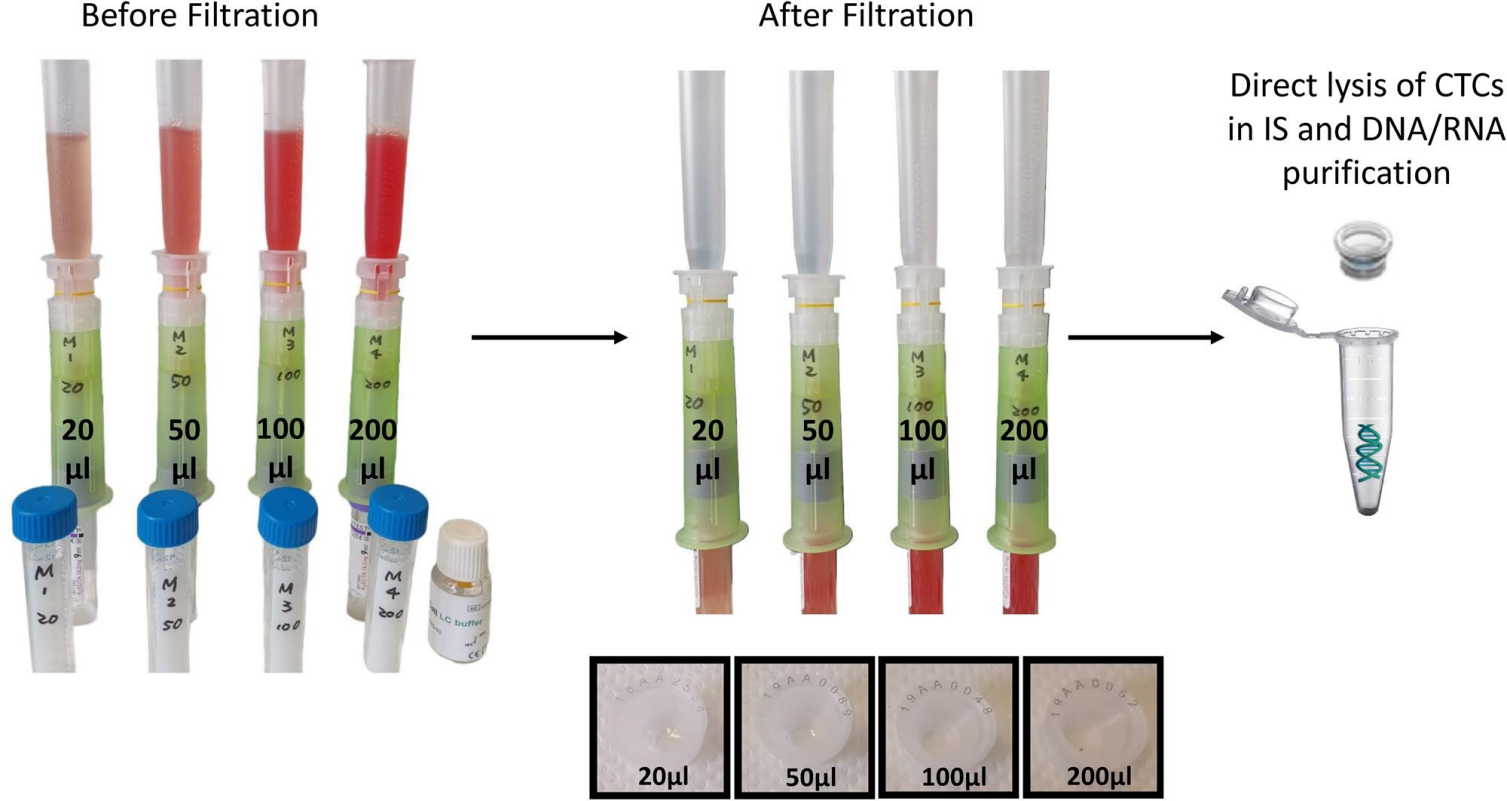
This figure depicts the successful filtration process of different volumes of mice blood using ScreenCell MB kits. The ISs are then inserted into 1.5 ml microcentrifuge tubes for further molecular biology analysis. This experiment was repeated 3 times.

In the next step of our experiments, we aimed to identify the optimal volume of mouse blood that is compatible with ScreenCell MB kits. Different blood volumes (20, 50, 100, and 200 µl collected in EDTA tubes) containing 0 or 50 4T1 cells were mixed with ScreenCell LC buffer and proceeded to filtration. This process was perfectly accomplished with all those conditions (Fig. 5). An attempt to treat higher blood volumes led to the failure of filtration and the blockade of the system (data not shown). Then, the ISs (capsules) of MB kits were inserted into 1.5 ml microcentrifuge tubes, and cells were directly lysed in the capsules. The extracted DNA was then collected in the bottom of the tubes. While the quantity of DNA was not in the detection range of current DNA measurement technologies for 20 and 50 µl samples, those of 100 and 200 µl samples had a mean DNA concentration range of 0.524 ng/µl. The following table depicts the DNA concentration for different conditions (Table 1).

**Table 1:** DNA concentration from WT mouse blood with or without spiked 4T1 cells isolated by ScreenCell MB device.

Mouse blood volume (µl)	Number of 4T1 spiked cells	DNA concentration (ng/µl)
100	0	0.503
100	50	0.600
200	0	0.446
200	50	0.550

The final elution volume is 25 µl. n = 2 for the experiments with 100 µl and n = 4 for the experiments with 200 µl of mouse blood.

### ScreeCell technology enables the culture of unaltered CTCs

**Figure 6:**
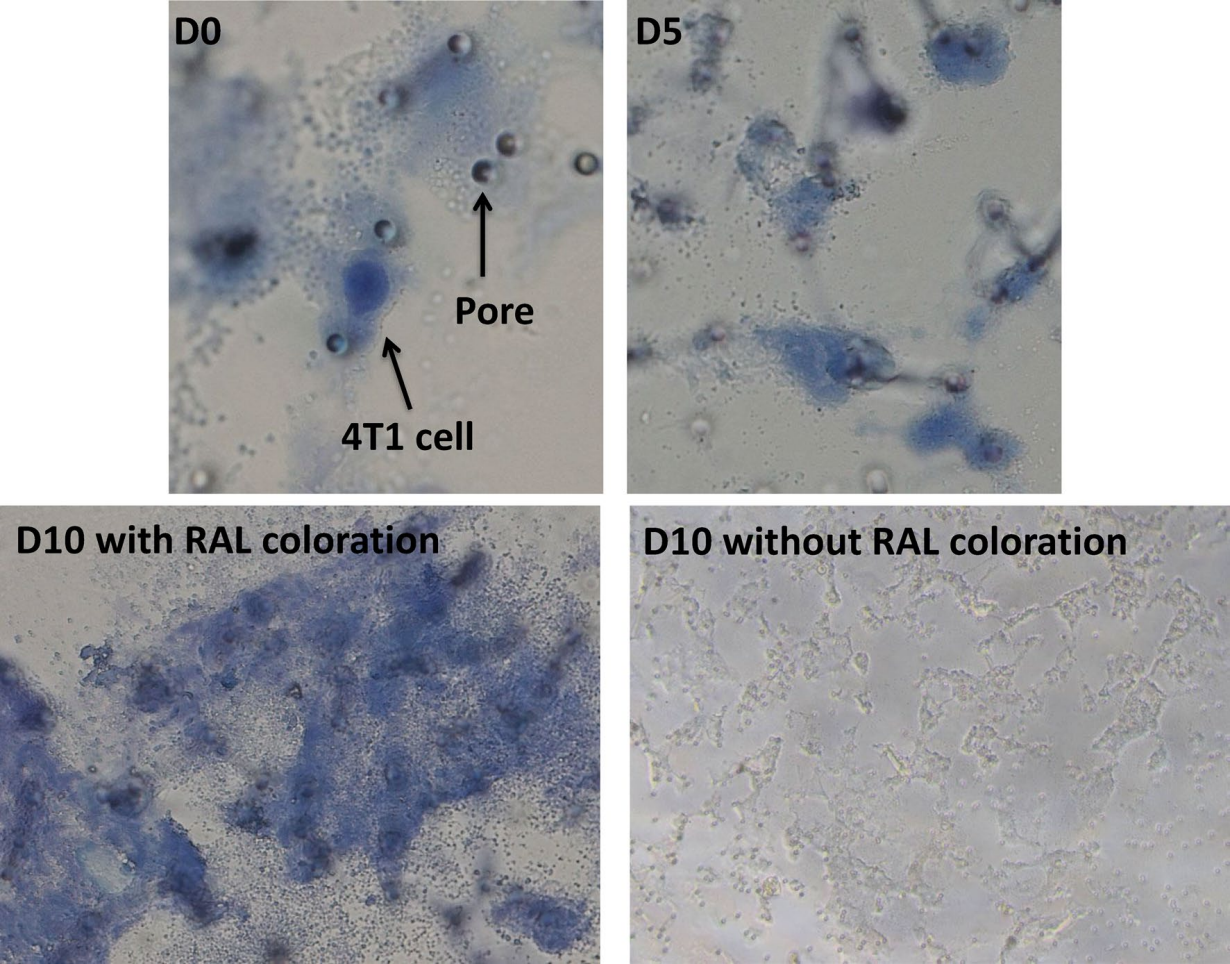
4T1 cells were spiked into WT mice blood and filtered with ScreenCell MB kits. ISs were then directly inserted into 24 well plates and cultured for 10 days. The images of D0, D5, and D10 with RAL coloration are taken using 40X, and D10 without coloration is taken with 20X magnification. This experiment was performed 4 times. D: Day.

Thanks to ScreenCell MB kits, CTCs are unaltered and could be grown in culture. In this final experiment, we spiked 500 4T1 BC cells into 100 µL of blood from healthy WT mice and proceeded to the filtration step. Then, ISs were immediately transferred to 24 well plates and kept in normal culture conditions for 10 days. Interestingly, isolated 4T1 cells were able to grow by forming cell colonies inside ISs during the culture (Fig. 6).

## Discussion

This study was designed to address different questions regarding the isolation and analysis of CTCs from mouse blood using ScreenCell technology. We have been able to validate K2-EDTA blood collection tubes as the most optimal choice for manipulating mouse blood. In particular, K2-EDTA has been also recommended as the anticoagulant of choice for blood cell counting and sizing by the International Council for Standardization in Hematology and the National Committee for Clinical Laboratory Standards (NCCLS).

Through examination of a series of different blood volumes, we have demonstrated that the ScreenCell Cyto kit could filter up to 500 µl and the ScreenCell MB kit up to a maximum of 200 µl of mouse blood. These values were the same for the Cyto kit and decreased to 100 µl for the MB kit after 24 h of blood preservation at 4 °C. This is indeed interesting since 200 µl represents around 10% of total mouse blood. Therefore, we suggest drawing 100 µl of blood in order to frequently repeat the desired experiments every one or two weeks according to the total body weight of tested animals without the need to sacrifice them^23^.

Moreover, we have tested two common models to investigate mouse cancer, i.e. transgenic mice with spontaneous tumors, or spiking human or mouse cancer cell lines into healthy WT mouse blood. In both settings, our results demonstrated a clear capture of both single and cluster CTCs or spiked cancer cells. This enables researchers to utilize a variety of mouse cancer models such as transgenic mice or the injection of cancer cells into immunodeficient or PDX humanized mice models^24^. Additionally, the isolated CTCs were morphologically distinguishable from normal epithelial cells and remaining leucocytes on the IS. Indeed, using the ScreenCell Cyto kit, one can go further with cytological and immunological analysis. FISH analysis and different immunostaining methods such as immunofluorescence (IF) and immunocytochemistry (ICC) are among the most common downstream analysis methods that are compatible with ScreenCell Cyto technology. Conventional immunostaining methods based on CD45 staining to target leucocytes and EpCAM and/or CK staining to target CTCs (in addition to cytological criteria) facilitate the enumeration of CTCs as the first crucial clinical information^25–27^. Furthermore, the expression of different informative antigens such as immune checkpoint molecules (PDL-1, CTLA4, etc.) and/or hormone receptors (estrogen, progesterone, androgen receptors, etc.) could be also evaluated.

The ScreenCell MB kits provide access to unaltered live cells, therefore, in our next step of experiments we extracted high-quality genomic DNA from isolated CTCs. We were able to show that 100 µl of mouse blood is the minimum volume required to have a detectable range of total extracted DNA and 200 µl is the maximum volume for this purpose. Indeed, the extracted DNA could be further analyzed for the detection of actionable or informative mutations such as EGFR, KRAS, NRAS, P53, PIK3CA, BRAF, etc. It is important to mention that the total extracted DNA contains a small fraction of CTC DNA. Therefore, highly sensitive downstream analysis methods such as NGS or digital PCR (dPCR) are necessary to detect the desired mutations. Indeed, using ScreenCell MB technology, we have already demonstrated the feasibility and compatibility of these two approaches. For instance, using the dPCR method, we have confirmed in patients with colorectal cancer that the detection of specific mutations such as KRAS is possible even in the presence of one CTC per milliliter of blood^28^. In another example, it was shown that using NGS, one can analyze specific mutations in CTCs of patients at a single cell level in metastatic BC^29^. Considering the emergence of precision medicine and the development of FDA-approved medications against specific mutations, CTC analysis could deliver real-time information about the validity of ongoing treatment and/or the potential appearance of novel targets over the course of cancer evolution.

Our final goal was to demonstrate the possibility of isolating live CTCs from mouse blood and growing them ex vivo. Our data using spiked 4T1 tumor cells showed the validity of this hypothesis since the isolated cancer cells continued to proliferate in the IS. In this study, we have used 4T1 cells which are very invasive and low-demanding cancer cells in culture. In fact, culturing real CTCs could be much more challenging as they might need specific growth factors, hypoxia conditions, and/or the presence of extracellular matrix elements^30^. The ability to grow CTCs could be especially interesting for drug development and pharmacological purposes since it provides a valuable source to test drug sensitivity and drug screening possibilities^31,32^. Ultimately, after testing the impact of new treatments, grown CTCs (with or without labeling) could be returned in vivo in order to evaluate their viability and metastatic properties.

## Conclusion

This work was designed to evaluate the compatibility of ScreenCell technology with mouse blood. Through a series of experiments, we demonstrated that ScreenCell Cyto and MB kits are compatible with mouse blood, especially in K2-EDTA tubes, and can filter up to 500 µl and 200 µl of blood, respectively. Additionally, unlike human blood, mouse blood samples could be kept in these tubes at 4 °C for 24 h. Using the ScreenCell Cyto kit, one can intuitively observe CTC cytomorphology and go further to downstream analysis. Thanks to the ScreenCell MB kit that captures the live unaltered CTCs, we successfully extracted and quantified their DNA or grew them in culture for at least 10 days. This provides a great possibility for their further pre-clinical analysis for precision medicine applications.

## Data Availability

The datasets used and/or analyzed during the current study are available from the corresponding author upon reasonable request.

## Abbreviations

BC: Breast cancer
CK: Cytokeratin
CTC: Circulating tumor cell
ctDNA: Circulating tumor DNA
EMT: Epithelial to mesenchymal transition
EpCAM: Epithelial cell adhesion molecule
EVs: Extracellular vesicles
IS: Isolation support
MB: Molecular biology
WT: Wild type
